# Understanding the Information Needs of Patients With Ovarian Cancer Regarding Genetic Testing to Inform Intervention Design: Interview Study

**DOI:** 10.2196/31263

**Published:** 2022-02-08

**Authors:** Yan Zhang, Siqi Yi, Ciaran B Trace, Marian Yvette Williams-Brown

**Affiliations:** 1 School of Information The University of Texas at Austin Austin, TX United States; 2 Center for Health Communication Moody College of Communication and Dell Medical School The University of Texas at Austin Austin, TX United States; 3 Department of Women’s Health Dell Medical School The University of Texas at Austin Austin, TX United States; 4 Department of Oncology Dell Medical School The University of Texas at Austin Austin, TX United States

**Keywords:** patient information needs, consumer health informatics, ovarian cancer, genetic testing, genetic counseling, mobile phone

## Abstract

**Background:**

Experts in gynecological cancer care recommend that all patients with invasive or high-grade ovarian cancer (OC) undergo genetic testing. However, even patients who intend to take or have taken genetic tests have many unaddressed information needs regarding genetic testing. Existing genetic counseling falls short of adequately addressing this challenge.

**Objective:**

This study aims to investigate the genetic testing–related information needs of patients with OC to inform the design of interactive technology-based interventions that can enhance communication of genetic testing information to patients.

**Methods:**

We interviewed 20 patients with OC who had taken genetic tests and gathered genetic testing–related messages from an active OC web-based community. The interview transcripts and web-based community messages were analyzed using the qualitative content analysis method.

**Results:**

Data analyses produced a comprehensive taxonomy of the genetic testing–related information needs of patients with OC, which included five major topic clusters: knowledge of genetic testing as a medical test, genetic testing process, genetic testing implications for patients, implications for family members, and medical terminology. Findings indicated that patients wanted to receive information that was relevant, understandable, concise, usable, appropriate, sympathetic, and available when needed. They also preferred various channels to receive information, including internet-based technologies, print, and conversations with health care providers.

**Conclusions:**

Patients with OC need a range of information to address the uncertainties and challenges that they encounter while taking genetic tests. Their preferences for channels to receive information vary widely. A multichannel information delivery solution that combines both provider-led and peer-to-peer education models is needed to supplement existing genetic counseling to effectively meet the genetic testing–related information needs of patients with OC.

## Introduction

Ovarian cancer (OC) is the second most common gynecological cancer in the United States [1]. Nearly 25% of OC cases are due to hereditary cancer syndrome as a result of breast cancer gene mutations (*BRCA1* and *BRCA2*) and Lynch syndrome [2,3]. The National Comprehensive Cancer Network and the Society of Gynecologic Oncology recommend that all patients with invasive or high-grade OC undergo genetic testing [4,5] as knowledge of gene mutations can inform targeted treatment [6] as well as cancer screening and prevention options for at-risk family members [7].

Nevertheless, the genetic test uptake rate among patients with OC falls short of expectations. For example, 2 studies reported that only 15% to 20% of all women diagnosed with OC underwent genetic testing for *BRCA1* and *BRCA2* [8,9]. Another more recent estimate of the testing rate among newly diagnosed patients with breast cancer and OC was 53% [10]. Although attention needs to be placed on promoting genetic testing uptake among patients with OC and their family members, there are unmet information needs among those who intend to take or have taken genetic tests that also need be addressed. For example, studies have reported that some patients with OC have never heard of *BRCA1* and *BRCA2*, are unaware of the relevance of genetic testing for themselves and their families, or underestimate the actual risk of a hereditary link to their diagnoses [6,11,12]. Studies have also found that some patients with cancer and patients at risk for cancer had concerns about genetic testing–associated risks, such as insurance discrimination, privacy infringement, and emotional distress [11,13,14].

Communication of information concerning cancer genetics and genetic services to patients needs to be improved to address patients’ literacy gaps and risk concerns to enhance patient satisfaction and sense of empowerment. Some interventions have been conducted [15-19]; however, most have focused on exploring noninferior alternative genetic counseling delivery models (eg, group counseling) to the traditional one-on-one face-to-face model, paying little attention to the materials delivered. Analyses of genetic counseling sessions have revealed that genetic counseling communication is largely provider-driven, centering on providing biomedical information and failing to consider patients’ information, communication, and psychosocial needs [15,20-27]. Furthermore, most interventions were delivered through traditional information channels (eg, booklets and telephone) or basic interactive technologies (eg, videos) [19], missing the potential that interactive web-based technologies can offer. Thus, there is significant room for designing web-based interventions to address patients’ genetic testing–related knowledge gaps and concerns.

Designing effective technology-based interventions requires a thorough understanding of patient information needs [28,29]. We define patients’ information needs regarding genetic testing as knowledge gaps that patients perceive or experience as preventing them from accomplishing genetic testing–related activities or goals. These knowledge gaps may result from cognitive and affective uncertainties and may be a result of environmental (including institutional, cultural, and societal) constraints [30-33]. Information quality (IQ), defined as “users’ perception of the quality of information presented on a Web site” [34], has been identified as a significant information-related factor that precedes the formation of people’s trust in and intention to use information systems [35-37]. The fulfillment of information needs is not possible if IQ is low. Thus, we also explore patients’ expectations of the quality of genetic testing–related information. In addition, we explore patients’ preferences concerning information delivery to fulfill our aim to inform system design. The specific research questions are: (1) Which topics of information do patients with OC need to be informed about regarding genetic testing? (2) How do patients characterize their preferences for the quality of genetic testing–related information? (3) From which information channels, media, or platforms do patients prefer to receive genetic testing–related information?

## Methods

Owing to limited research on this subject, we adopted a qualitative research design consisting of two methods: interviews and analyses of web-based community posts.

### Interviews

#### Participant Recruitment

The participants were women who had been diagnosed with OC and had undergone genetic testing. Recruitment was performed in 3 ways. The first was a chart review by a clinical research assistant at the Dell Medical School at the University of Texas (UT). More than 30 eligible patients who received treatment from a physician in the LIVESTRONG Cancer Institutes at the school were contacted. Reasons for not participating included a lack of interest or energy, language barriers (non–English-speaking), and a lack of resources (car, computer, or webcam). Second, we posted email recruitment messages to the mailing list of the National Ovarian Cancer Coalition Austin and San Antonio Chapter. Third, we adopted word-of-mouth and snowballing recruitment strategies. Recruitment efforts using all 3 venues spanned the entire research process (data collection and analysis) and halted when a theoretical saturation of the data was observed. The data were deemed saturated when no new genetic testing–related information needs, IQ, or information delivery themes emerged from the data. A total of 20 patients with OC participated in the interviews, of which 8 (40%) were recruited through the chart review, 10 (50%) were recruited through the mailing list, and 2 (10%) were recruited through word-of-mouth.

#### Interview Design

The interview protocol had three components: a demographic questionnaire, a semistructured interview, and a co-design session. The guide for the interview and the co-design session is provided in Multimedia Appendix 1. The demographic questionnaire collected the participants’ background information, including demographics (eg, age, race, ethnicity, and education), cancer diagnoses, and genetic test results. In the semistructured interviews, the participants recalled their genetic testing process (from when they were prescribed the test to receiving the test results) and experience (including motivations, emotions, interactions with health care providers and family and friends, and challenges). They were also asked to describe their genetic testing–related information behaviors, including information needs, information sources, and information-seeking efforts.

**Figure 1:**
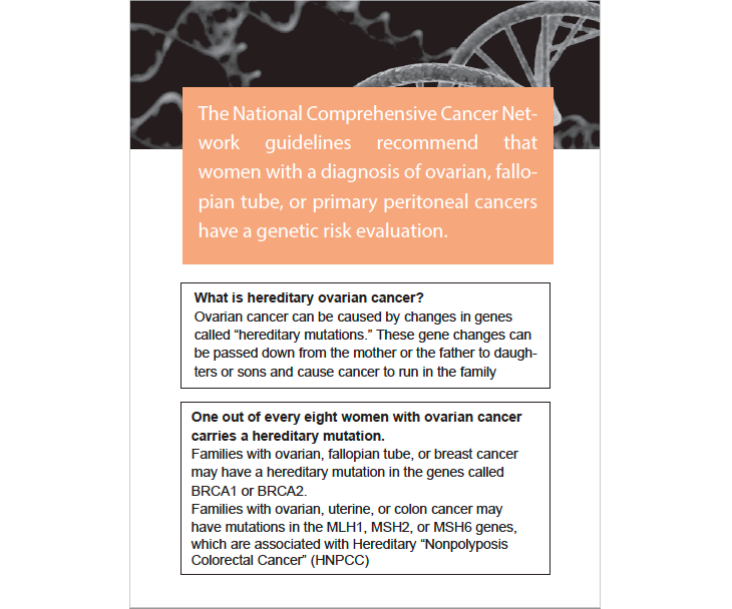
A sample page of the paper mockup. BRCA: breast cancer gene; MLH: mutL homolog.

**Figure 2:**
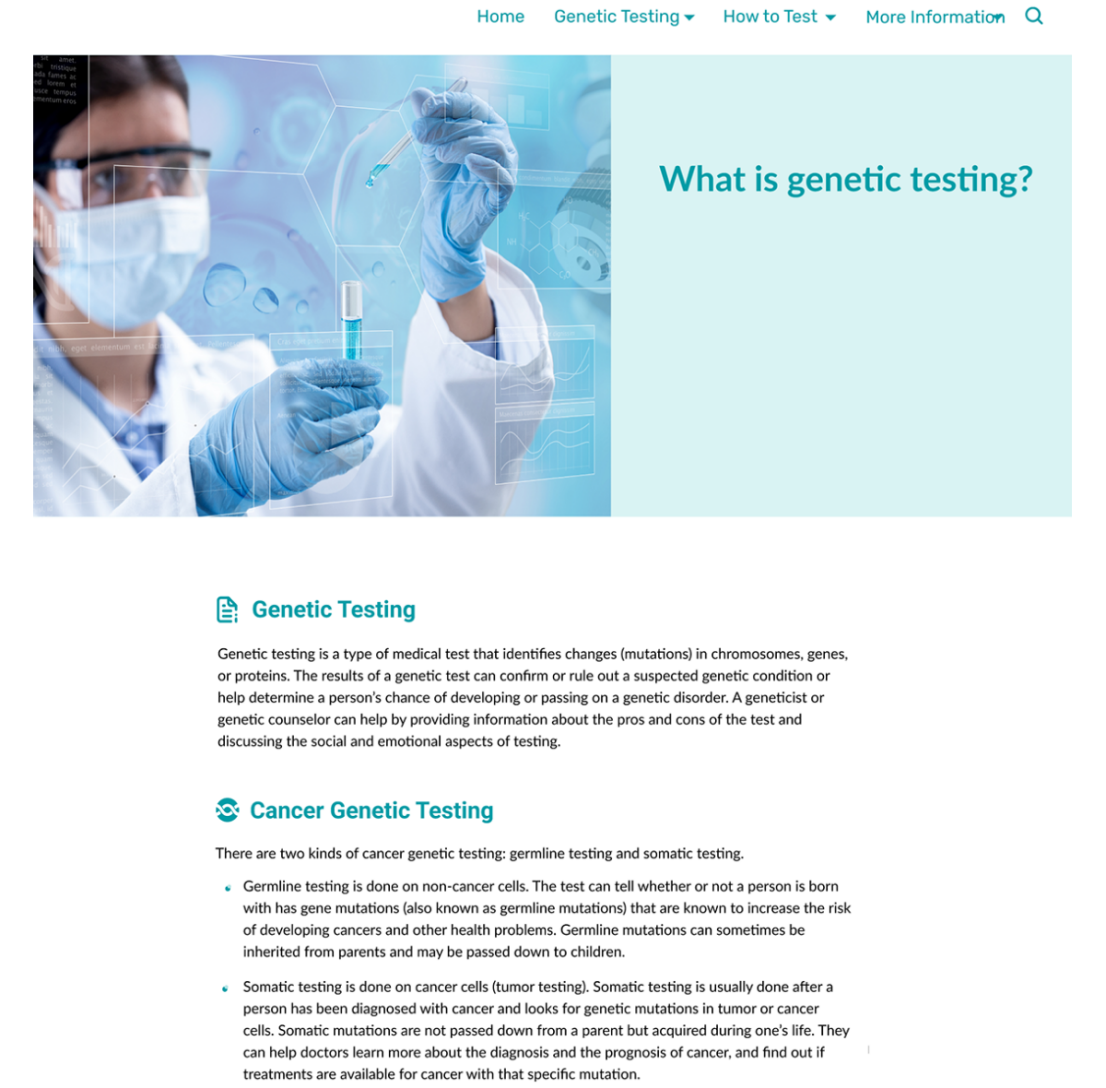
A sample webpage of the digital mockup website.

In the co-design session, the participants reviewed and commented on a mockup website that offered genetic testing–related information while imagining that they were co-designing the website for patients such as themselves. They were also asked to describe any additional content that they thought should be included on the website, their expectations of IQ, and how they wanted genetic testing–related information to be presented and delivered to them. The co-design session was used because people sometimes experience difficulty in perceiving and articulating their information needs and preferences [38]—interactions with information sources may make some information needs and preferences for IQ and information delivery more visible [39]. Questions concerning IQ were framed based on a successful validation of the information system success model by DeLone and McLean [29], which identifies six IQ dimensions: availability, usability, understandability, relevance, format, and conciseness [40,41]. The initial mockup was paper-based, created based on a review of studies on the genetic testing–related information needs of patients with OC (Figure 1). The paper mockup was later developed into a digital mockup (Figure 2) based on ongoing analyses of the interviews. The content displayed on the mockups was drawn from trustworthy sources such as the National Cancer Institute and the Centers for Disease Control and Prevention.

#### The Interview Process

The interviews were conducted between February 2019 and October 2020. The first 6 participants (6/20, 30%) were interviewed in 5 face-to-face focus groups that took place in a private conference room at the UT campus. Each focus group consisted of 2 participants. A total of 2 participants (2/6, 33%) took part in 3 focus groups as we were not able to complete both the interviews and the co-design activities in 1 session. The other 4 patients (4/6, 67%) participated in 1 focus group session each. Upon arrival, researchers greeted the participants, gave them an introduction to the project, and asked them to review the consent form. The participants were encouraged to ask clarifying questions when needed. After providing consent, the participants completed the demographic questionnaire. They were then interviewed about their genetic testing process and experience as well as genetic testing–related information-seeking activities. The focus group interview format was adopted as it allows for interactions between participants with the goal of helping participants recall and elaborate on their genetic testing experience. The interviews were followed by the co-design session. Upon completion of the co-design session, the participants received a US $30 Amazon gift card.

Owing to the COVID-19 pandemic, the subsequent 13 interviews (13/20, 65%) were conducted one-on-one through the Zoom web conferencing platform, and 1 participant (1/20, 5%) was interviewed through emails. In these interviews, the participants completed the consent process and the background questionnaire on the web on Qualtrics (Qualtrics International Inc) before the interview. The Zoom interviews followed the same procedure as the focus groups. For the email interview, we sent the questions to the participant, and she responded with written answers. In total, 2 researchers (YZ and SY) reviewed and discussed the answers and then asked clarifying questions by commenting on the answers. She then replied to the clarifying questions. A total of 3 rounds of email correspondence took place. The URL of the digital mockup website and the questions that we asked in the co-design session were then emailed to the participant. She answered those questions. Similarly, we asked clarifying questions by replying to her answers.

The focus group and Zoom interviews lasted 40 minutes to 2 hours. Each interview was conducted by at least two researchers, audio-recorded, and later transcribed. The researchers held a 20- to 30-minute debriefing session after each interview to generate main themes related to the research questions and insights to inform the design of the digital mockup website.

### Web-Based Forum Message Analysis

Social media platforms (eg, web-based health forums and social question and answer platforms) are sources for collecting authentic consumer health information needs [42]. Web-based posts are also considered an ecologically valid means of eliciting user needs for technological design [43]. We searched the OC community on the American Cancer Society’s Cancer Survivor Network (CSN) to identify genetic testing–related posts. The keywords used for the search included *genetic testing*, *genetic counseling*, *BRCA*, and *DNA testing*. The search identified 210 messages. We manually collected these messages, read them, and, of the 210 messages, we retained 25 (11.9%) that contained patients’ genetic testing–related information needs for analysis. These messages were posted by 25 unique IDs between December 2008 and June 2018. Excluded posts included answers to the questions posted, genetic testing resources, the patients’ own OC experiences, and family members’ concerns for themselves.

### Data Analyses

The interview transcripts and CSN messages were analyzed using both inductive and deductive approaches to the qualitative content analysis method [43]. First, we imported the interview transcripts and web-based forum messages to MAXQDA 2018 (VITERBI Software GmbH), a qualitative data analysis software. Initially, YZ coded 5 interview transcripts deductively by following the definition of information needs (ie, knowledge gaps that patients perceive or experience as preventing them from accomplishing genetic testing–related activities or goals) and the IQ dimensions outlined in the study by Petter et al [41] (including availability, usability, understandability, relevance, format, and conciseness). An inductive approach was then applied to generate subcategories of genetic testing–related information needs, additional categories of IQ dimensions, and technological platforms for information delivery [44,45]. A codebook was developed to keep track of and explicate the coding system.

CT and SY applied the codebook to independently code 2 interview transcripts. The research team then held several collaborative coding sessions to discuss codes, paying special attention to reconciling codes to reduce overlap and redundancy between subcategories [45]. This effort resulted in a revised codebook. SY, CB, and YZ each revisited the codes that they had assigned to the transcripts by applying the new codebook. Each researcher then coded a subset of the remaining transcripts. SY coded the forum posts. All codes were validated by a different coder to enhance coding reliability, and disagreements were resolved based on discussions between all research team members.

### Ethics Approval

The University of Texas at Austin Institutional Review Board approved the study.

## Results

### Interview Participants

Most participants were aged >40 years (19/20, 95%), White (16/20, 80%), non-Hispanic (13/20, 65%), and had a college or postgraduate degree (13/20, 65%; Table 1). Their cancer stage at diagnosis varied. Of the 20 participants, all of them (100%) had previously undergone germline genetic testing, and 6 (30%) had also undergone somatic genetic testing. Most of these tests (13/20, 65%) were conducted in the past 3 years (2018-2020). The test results varied.

**Table 1:** Participant characteristics (N=20).

Characteristics		Participants, n (%)
**Age (years)**		
	<40	1 (5)
	40-49	6 (30)
	50-59	5 (25)
	60-69	6 (30)
	70-79	2 (10)
**Race or identity**		
	White	16 (80)
	American Indian or Alaska Native	1 (5)
	Mexican-American	1 (5)
	Not reported	2 (10)
**Ethnicity**		
	Hispanic or Latino	7 (35)
	Non-Hispanic or Latino	13 (65)
**Level of education**		
	<8 years	1 (5)
	8-11 years	1 (5)
	Post–high-school training other than college	2 (10)
	Some college	3 (15)
	College graduate	7 (35)
	Postgraduate	6 (30)
**Cancer stage when diagnosed**		
	1	3 (15)
	2	3 (15)
	3	6 (30)
	4	5 (25)
	Not reported	3 (15)
**Year in which the most recent genetic test was taken^a^**		
	2018-2020	13 (65)
	2015-2017	5 (25)
	2012-2014	2 (10)
**Test results**		
	Germline positive	6 (30)
	Germline negative	7 (35)
	Variants of uncertain significance	1 (5)
	Germline negative and somatic positive	1 (5)
	Germline and somatic negative	5 (25)

^a^All 20 participants had taken a germline test, and 6 (30%) had also taken a somatic test.

### Information Need Topics

Patients’ genetic testing–related information needs coalesced around five topic clusters: basic knowledge of genetic testing as a medical test, genetic testing process, implications of genetic testing for patients, implications for family members, and medical terminology.

#### Basic Knowledge of Genetic Testing as a Medical Test

The cluster of basic knowledge of genetic testing included two topic categories: basic features of genetic testing and standards and regulations (Table 2). Example questions were extracted from the data and rephrased for conciseness and clarity.

Regarding genetic testing features, the patients wanted to know what genetic testing is, what it does, and its benefits and potential risks. In terms of standards and regulations, the patients wanted to learn who approves genetic testing, who is qualified to provide it and the providers’ qualifications, and relevant government standards and regulations.

**Table 2:** Patient information needs regarding basic knowledge of genetic testing (GT) as a medical test.

Category and subcategory		Example questions
**Basic features of GT**		
	What is GT?	How are clinical forms of GT different from direct-to-consumer GT? (I^a^) What are the distinctions between the different methods of GT recommended for cancer patients? (C^b^) What are the current tests for ovarian cancer? (I)
	What does GT do (ie, functions)?	What does GT do? (C) What does GT uncover or look for? (I) Can GT determine or test for cancer? (I) Can GT determine other diseases? (I) How is the information from GT used? (I)
	Benefits (why GT?)	Why would you want to be genetically tested? (I) Why is GT important? (I) How is GT beneficial in saving lives and helping families get pre-screening to detect cancer sources? (I) What benefits can GT results bring to the treatment of OC^c^? (I, C)
	Risks	What are the possible risks of GT? (I)
Standards and regulations		Who approves GT? Is the FDA^d^ involved? (I) Who is qualified to offer this service? What are their qualifications? (I) What are the standards and regulations related to GT? (I)

^a^Indicates that the example is from the interviews and co-design sessions.

^b^Indicates that the example is from the web-based community message analysis.

^c^OC: ovarian cancer.

^d^FDA: Food and Drug Administration.

#### Genetic Testing Process

This topic cluster included three topic categories: financial demands, taking the test, and obtaining results (Table 3).

Cost was often the patients’ first concern when considering taking genetic tests. As an interview participant put it, “cost was my first question.” They wanted to know whether their insurance covered the test and, if not, how much they must pay. In several cases, the participants did not have insurance, and third parties (eg, foundations) subsidized the cost. A few participants mentioned that they had considered not taking genetic tests if the cost was not covered by insurance or third parties.

**Table 3:** Information needs regarding the genetic testing (GT) process.

Category and subcategory		Example questions or comments
**Financial demands**		
	Cost	What is the cost of GT? I would want to know the costs right up front. (I^a^) Do I have to pay for GT? (I) The hard thing I've noticed in the US is that they often can't tell you even how much your cost is, because it depends on your insurance and all these ridiculous things. (I)
	Insurance coverage	I would want to know whether my insurance covers GT. (C^b^, I) Does Medicare cover GT? (C)
**Taking a GT**		
	Who does GT?	Can a regular doctor perform GT for cancer genes? (C) Who is doing the test? Who are they? (I) Who are the testing companies? Can we choose which one to use? (I) What lab will you need to go to take the test? (I)
	Procedure and test details	I would be interested in knowing how GT is done and have a better understanding of that. (I) What exactly happens in the lab? (I) How much blood will be drawn? Is there an alternative to a blood draw? (I) Is it painful? (I)
**Obtaining results**		
	Receiving GT results	What is the timeframe for getting GT results? (I) Will I be contacted when they find new information from the test results? If so, how, and when will I be contacted? (I) Can I get a copy of the GT results? (I)
	Genetic counseling	How long do I have to wait to see the genetic counselor? (C) What questions should I ask during a genetic counseling session? (C) I’m not sure whether or not to have my GT results interpreted. (C) Who would I talk to about GT to understand if my ovarian cancer was genetic or not? (C) Who will interpret the results for me? (I) What is the significance of a particular result, like VUS^c^? (I)

^a^Indicates that the example is from the interviews and co-design sessions.

^b^Indicates that the example is from the web-based community message analysis.

^c^VUS: variants of uncertain significance.

Regarding taking the test, the patients wanted to know who recommends and orders genetic testing, who conducts genetic testing, the testing companies involved, and the laboratories that perform the test. They also wanted information about test procedures, including how it is done, whether a blood draw is needed, and whether it is painful.

Information about when and how they receive the test results and whether they can obtain a copy of the results was also needed. Some patients knew about genetic counseling and asked specifically about it, including when to receive genetic counseling and what questions to ask. Some patients hesitated to pursue genetic counseling and sought peers’ opinions (through web-based communities). Some patients were not aware of genetic counseling and wondered who could help interpret their genetic test results.

#### Implications of Genetic Testing for Patients

##### Overview

This topic cluster included five topic categories: cancer causes, clinical implications, genetic discrimination, lifestyle, and communication with family (Table 4).

**Table 4:** Information needs concerning the implications of genetic testing (GT) for patients.

Category and subcategory			Example questions or comments
Cancer causes			Do I have a genetic mutation? Am I a carrier? (I^a^) I was curious to see if I had a genetic mutation for the cancer to begin with. (I) What caused my cancer? Genetic mutation or my diet? (I) My GT result indicates that I am at risk for breast cancer, but I had ovarian cancer, not breast cancer, I need an explanation. (I)
**Clinical implications**			
	Treatment	Can GT results affect my cancer treatment? If so, what are the effects? (I, C^b^) What type of chemotherapy do you get if positive for a BRCA^c^ mutation? (C) Will I have a harder time fighting off the cancer given that I have tested positive for the BRCA2 mutation? (C)	
	Preventative strategies to reduce cancer risks	What preventative measures can be done if the results come out positive? (I, C) How do I know if I should follow the doctor’s advice regarding preventative surgery? (C)	
**Genetic discrimination**			
	Insurance discrimination	Does anyone know of cases of insurance companies using a GT result to deny benefits to subscribers? (I) Could the GT result be used against me to deny my healthcare or life insurance coverage? (I, C) Is this going to affect my insurance later in my life? Am I going to have to pay more money somehow? (I) Who has access to my GT information? Are there laws to protect us from genetic discrimination [vis-à-vis health insurance]? (I, C)	
	Employment discrimination	Can my GT result records be used to deny my employment? (I)	
Lifestyle			Is there anything I can do in relation to lifestyle and diet to minimize any problems that might rise from the GT being positive for a mutation? (I) If my genetic testing is abnormal, are there lifestyle or diet modifications that are helpful to reduce the risk of developing cancer? (I)
Communication with family			I was worried like if I had genetic mutations, at what point do I discuss this information with my children? (I) How do I approach my family and talk to them about GT results? (I, C)

^a^Indicates that the example is from the interviews and co-design sessions.

^b^Indicates that the example is from the web-based community message analysis.

^c^BRCA: breast cancer gene.

##### Cancer Causes

The patients showed a great deal of interest in seeking answers to the following question—*what has caused my cancer*—in light of their genetic test results. When they had mutations related to breast cancer but not OC, they wanted explanations for why they had developed OC. When the genetic test results were negative, some patients questioned whether it was their lifestyle (eg, diet) that caused the cancer.

##### Clinical Implications

Questions concerning clinical implications mainly focused on 2 aspects. The first was how the results can inform treatment. Questions ranged from general inquiries about whether test results would affect the treatment to questions about specific therapies. For example, a patient posted the following on the CSN community:

Within the last couple of days there was new information about BRCA women who had ovarian cancer (I think BRCA2 not sure) and new chemotherapy available for that. Has anyone else who has ovarian cancer gone for BRCA testing? If so, what type of chemo did you get?

The second aspect was what preventative measures could be taken to reduce the risk of other cancers, mostly breast cancer. Questions ranged from general inquiries about what preventative measures are available to more specific inquiries about preventative surgeries (eg, prophylactic mastectomy). The following message from the CSN community is an example:

[Has] anyone had to undergo a prophylactic mastectomy to PREVENT breast cancer? I have tested positive on genetic testing after stage 3 ovarian cancer and now [doctors are recommending] the mastectomy. Have many questions!

##### Genetic Discrimination

Patients were concerned about who has access to their data, whether the data could be used to deny them health or life insurance or raise insurance costs, and whether there are laws to protect them from such discrimination. Worries about potential employment discrimination were also expressed.

##### Lifestyle

In relation to lifestyle, patients expressed a need to know how they can modify their lifestyle (eg, diet) to minimize risks incurred by genetic mutations and to manage treatments.

##### Communication With Family

As a patient’s post on the CSN community illustrated, *“*it is a horrible thing to have to tell your family members they [too] might [develop cancer]*.*” Some patients expressed a need to gain knowledge about how and when to talk with family members about their genetic test results, particularly if the results were positive for a mutation.

#### Implications of Genetic Testing for Family Members

##### Overview

Patients’ information needs regarding genetic testing implications for family members focused on family members’ cancer risks and on concerns regarding insurance discrimination and emotional distress (Table 5).

**Table 5:** Information needs regarding the implications of genetic testing (GT) for family members.

Category and subcategory		Example questions or comments
**Cancer risks**		
	GT screening	Who (which family members) should be tested? (I^a^, C^b^)
	Prevention and monitoring strategies	What course of action can be taken [for family members] if I tested positive? (I, C)
Insurance discrimination		Will my family members be denied insurance? (C) How would positive results affect my children when they need their own healthcare? (I)
Emotional distress		Will my family be living in fear as a result of positive GT results? (C) I worry that my family will be living in fear. (C)

^a^Indicates that the example is from the interviews and co-design sessions.

^b^Indicates that the example is from the web-based community message analysis.

##### Cancer Risks

Information concerning cancer screening for family members was a category of information needed. For example, an interview participant noted the following:

When my test returned as positive. I have only one concern. I worry about passing [the genes] to my kids.

She later added that “the next [question] is who should be tested?”

Some patients also wanted to be informed of cancer prevention and monitoring strategies (eg, surgeries) that family members can follow if their genetic test results are positive. For example, an interview participant said the following:

[My niece] had 3 children and she’s done having children. Her genetic makeup is kind of similar to ours, and probably that would be something she could have monitored easily and if she did carry that and was concerned, she could have her ovaries removed before she had any problem. I think if you find you are predisposed of having breast cancer, there are somethings you can do to minimize your risk. My sister is correct that knowledge is power.

##### Insurance Discrimination

Some patients worried about insurance discrimination against their family members. The following post on the CSN community demonstrates this concern:

I had the genetic testing in March and some of my family members were [leery] of

being denied insurance benefits

##### Emotional Distress

Some patients worried that their genetic test results may cause stress to their family members. For example, a patient commented the following on the CSN community:

I have a lot of cousins, and none have gotten cancer even though most of us are in our 50s. I certainly would hate to think of my 2 daughters (ages 14 and 22) having to suffer from cancer. I wouldn't want them to feel afraid of that. So, it is unlikely I would do any sort of genetic testing.

#### Terminology

The need to understand genetic testing–related medical terminology cuts across different stages of the genetic testing process. A CSN community user mentioned the difficulty of articulating requests for genetic testing:

I want to call my doctor to give me a written request for [cancer] genetic testing. What should I ask for? Can't seem to find the exact terminology on the [Internet] and I want to be sure it's correct.

An interview participant called such terms “the big words” and mentioned difficulties in understanding genetic test results:

I looked up [online] some of the words [in my GT results] to see what they mean. I don’t know any of them.

### Patients’ Preferences Concerning IQ

The participants expressed preferences for seven IQ dimensions: relevance, understandability, conciseness, usability, appropriateness, being sympathetic, and availability (Table 6).

**Table 6:** Patient preferences concerning information quality.

Information quality dimension			Example participant comments
Relevance			“[A website is of interest to me when it is about] BRCA^a^ [and] linked to ovarian cancer.” [Participant 18]
Understandability			“The basics are good. The nurses break it down to basics and to my level.” [Participant 2] “I wish they would have just broken it down in layman terms for middle aged women that aren’t so tech savvy. Just simple, simple words.” [Participant 18]
Conciseness			“I think your text is informative, but not overwhelmingly long. It’s short and concise and to the point.” [Participant 14] “People may be fearful to look at something that’s a little more detailed.” [Participant 17]
**Usability**			
	Tables, bullets, and white spaces	“Maybe a table would help. Genetic drives of cancer. There is a lot of good statistics in there...I know I tend to look at tables and statistics.” [Participant 17] “I like it because it’s nice and clean and has a lot of white space and bullets.” [Participant 9] “I really liked that you have a lot of white space, you know, on the page because I think that that helps make it less intimidating.” [Participant 18] “There’s a fair amount of space. I mean, it’s not overloaded.” [Participant 15]	
	Additional sources	“I think...providing basic information and with links to find out more. Someone wants to kind of expand on that basic information.” [Participant 2] “...have the ability to go deep or stay high.” [Participant 16]	
Appropriateness			A comment on an image used on the mockup webpage about test results: “She looks very happy for having such a serious conversation. She just looks just a little too happy for that. I mean, it, as I remember, it was, it was stressful, not horrifically stressful, but it was stressful waiting for the results.” [Participant 16] “I don’t know if I’d want to show [a picture that shows tubes containing blood] just because of those few people I’ve met that are so fearful of blood.” [Participant 13]
Being sympathetic			“...what would get my attention would be if there was something that said, Hey, you don’t have to have cancer [to get genetic testing]. Don’t be afraid of this. It’s not a death sentence. It’s not, you know, you’re looking into a crystal ball or having someone read your future.” [Participant 18]
Availability			“[The mockup website] probably would have been a comfort to me to be able to go and look these things up. And just because so many times in the beginning, I found myself going back over the same stuff over and over, what does this mean? What does this mean? And I think, well, I already read that, but did I miss something when I read it.” [Participant 8] “It’s something that, you know, that you can take with you, especially cause when you’re, you’re going somewhere and all of a sudden you have a question about, well, was that really what I thought it was and you can go back and look at it.” [Participant 17]

^a^BRCA: breast cancer gene.

*Relevance* refers to the information provided being directly relevant to OC-related genetics and genetic testing. *Understandability* refers to whether the information is easy to understand. The participants used terms including “basic,” “simple,” “self-exploratory,” “straightforward,” “layman’s terms,” and “easy to digest” to express this expectation. *Conciseness* indicates that the information should be brief and succinct. Too much detail may incur a sense of information overload and discourage some patients from reading further.

*Usability* indicates that the information should be user-friendly. In this study, the concept was mostly related to the information presentation format. The participants preferred structured formats—tables, bulleted lists, and white spaces—as they made the text less “intimidating” and were easier to follow. The participants weighed usability over the amount of information they could receive. They suggested the use of hyperlinks to expand beyond basic information when needed.

*Appropriateness* was mainly about the images used in this study. The participants expressed concerns about several images on the mockup website, commenting that they instilled fear or were inappropriate for cancer contexts (eg, one image showed tubes containing blood and the other image showed a character with a seemingly happy smile that was perceived to be unfit for a medical consultation setting). Being *sympathetic* suggested that the participants wished that the information had an understanding and encouraging tone, showing consideration of information seekers’ emotional states (eg, fear and need for hope). *Availability* represented the participants’ expectations that the information source would be available for them to access whenever and wherever needed.

### Patients’ Preferences Concerning Information Delivery

Table 7 shows the participants’ preferences for channels from which to receive genetic testing–related information.

**Table 7:** Patient preferences concerning information delivery.

Channel and subcategory		Specifics
**Digital technologies**		
	Media and platforms	Internet Websites Email Mobile apps Patient portals Social media
	Devices	Computers (laptop or desktop) Smartphones Tablets
Paper-based prints		Pamphlet or brochure Written information to take home
Health care providers		Gynecologist Oncologist Nurse navigators Nurses Genetic counselors Insurance company Genetic testing company

Some participants preferred to receive information from digital technologies, varying from the internet (in general), websites, and email to patient portals, social media, and mobile apps. Some participants valued the social interaction affordances offered by certain digital technologies. For example, participant 12 suggested the following:

Probably on a website, even on an app. I mean, because you know, it wasn't until I was diagnosed with cancer that I realized there's so many apps out there that talk to other people going through what you're going through...And they post like what they're going through, what kind of meds they're on, what kind of chemo they took. And it kind of makes you understand what other people are going through. And so, it kind of helps you, and then you know if there was something like that too [about GT], and that would help person.

Their preferred devices for accessing information also differed and included laptop or desktop computers, smartphones, and tablets. The participants saw a need to make the information source adaptive to these different screen sizes, as participant 7 suggested:

Just make sure it is mobile friendly as you don’t know if folks will access it via a desktop computer, laptop, tablet or their smart phone.

Some participants preferred to receive pamphlets, brochures, or some other form of written information to take home. For example, participant 16 indicated the following:

I would want it printed. Okay. I'm still old school...in spite of designing computer systems for a living. I still like paper.

Participant 17 commented the following:

I think something that you can save I think written is good.

Other participants preferred to receive genetic testing–related information directly from health care providers, including gynecologists, oncologists, nurse navigators, nurses, genetic counselors, insurance companies, or genetic testing companies. For this channel, the preference was for information to be conveyed through face-to-face meetings, phone calls, or written materials such as pamphlets. For example, participant 12 described the following:

Well, I've been seen at a gynecologist since I was. I think the very first time I went to go see a gynecologist, I was maybe like 23 or 24. And I had never heard of genetic testing until when I got diagnosed with the cancer. So, I think somewhere in between, you should be told, you know, Hey, get this, you know, you might help me. You know, I, cuz I know like, like when my niece was in her teens, they were offering that shot for the cervical cancer. I don't remember what it's called. Yes. It wasn't there when we were, when we were growing up, it's something fairly new. And I think that would probably have helped many people along the way, you know? So, anything that could prevent something like this, I think is good.

Participant 17 described that she expected to receive genetic testing–related information from a nurse or a staff member in oncologists’ offices:

I think someone separate would actually be better because I think that really, and truly the doctors are trying so hard to save your life, that you get super focused in on that. And I think, I think someone like maybe a nurse maybe just a certain staff member at the doctor's office.

## Discussion

### Principal Findings

This study investigated the information needs of patients with OC related to genetic testing and their preferences for IQ and information delivery to inform interventions to enhance the genetic testing experience and sense of empowerment of patients with OC. This makes 3 major contributions to the literature, as detailed below.

#### Taxonomy of the Genetic Testing–Related Information Needs of Patients With OC

Previous studies on the genetic testing–related information needs of patients with OC are limited. They have mostly used the survey method [11,46,47] and focused on genetic counseling instead of the patients’ entire genetic testing process [12], limiting the range of information needs identified. We explored patients’ information needs throughout their genetic testing process, from when they were prescribed the test to when they received and reflected on the test results, using multiple qualitative methods, including interviews, participatory co-design activities, and the analysis of genetic testing–related messages on an active OC web-based community. Together, these methods afford in-depth inquiry of the information needs of patients with OC, leading to a comprehensive taxonomy of their genetic testing–related information needs. This taxonomy confirmed many genetic testing–related information needs of patients with breast cancer and OC reported in previous studies, such as the purpose of testing, implications for treatment decisions, treatment options, time frame for results, and the availability of predictive testing for relatives [11,12,14,15,46-49]. It also revealed numerous topics that have been less reported in the literature, such as genetic testing–related standards and regulations, financial demands, medical professionals involved in genetic testing, communication with family members about genetic testing, and the impact of genetic test results on patients’ lifestyle [15].

Many of the needs identified in the taxonomy are consistent with expert genetics and cancer health professionals, who agree that information about inheritance, cancer risks, and management are key messages for patients with cancer [50]. Clinical guidelines for genetic counseling also recognize that some topics in the taxonomy should be covered in pre- and posttest genetic counseling, such as psychological issues, including coping with disclosure of test results, and social issues, including the impact of testing on insurance, employment, and family relationships [51]. Nevertheless, it is still important to recognize patients’ perspectives and priorities regarding their own information needs, considering that patients continue to report various unmet needs years after the release of clinical guidelines for genetic counseling [11,12,14]. Thus, this taxonomy can serve as a patient-centered road map for creating information architectures for interventions that address the information needs of patients with OC.

It is also important to acknowledge the limitations of the taxonomy in light of the methods we adopted. The semistructured interview and participatory co-design methods afford the ability to delve deeply into a set of issues, probe and ask follow-up questions, and connect ideas in real time as a discussion unfolds; however, the methods assess information needs retrospectively, increasing the chance that the participants might not have recalled all the information needs that came up before, during, and after the genetic testing process. The web-based community message analysis can help compensate for the limitations of the interviews as the messages represent patients’ real-time information needs; however, the number of posts that we were able to collect was constrained because of a lack of discussion on this topic among users of the chosen web-based community.

#### IQ as an Attribute of Information Needs

Guided by the information system success model by DeLone and McLean [29], we identified seven IQ dimensions that patients with OC deemed important: relevance, understandability, conciseness, usability, appropriateness, being sympathetic, and availability. This finding is consistent with the finding of previous empirical studies that patients with cancer prefer brief, straightforward, personalized, and positive information for genetic testing communication [6,47]. Nevertheless, we examined patients’ IQ preferences from a more systematic approach (ie, both theory- and data-driven). These IQ dimensions together offer insights on how information should be written, organized, and presented so that it is more likely to be used by patients, supplementing the insights offered by the information needs taxonomy and providing important guidance for intervention design. Previous studies have measured attributes of consumer health information needs, including level of importance [52], extent of fulfillment [53], amount of information needed [54], and frequency [55], but have largely ignored users’ IQ expectations. Our research results suggest that, as an information-related factor that significantly affects system adoption and success [37], IQ should be considered as an important attribute to successfully address patients’ information needs.

Theoretically, the results suggest that the model by DeLone and McLean, despite being developed and tested mostly in organizational settings, was effective in guiding the exploration of IQ desired by patients with OC as all quality dimensions specified in the study by Sedera et al [40] were found in our data (format was integrated with usability). However, two new dimensions—appropriateness and being sympathetic—emerged from our research. Both dimensions attend to people’s emotional states and may be context-specific as most of the participants mentioned that genetic testing occurred during a chaotic and uncertain time when they were busy coping with a cancer diagnosis and dealing with treatment. Efforts are needed to theorize the impact of health information needs and information-seeking contexts on consumer IQ expectations.

#### Information Delivery

Previous studies have reported that patients with OC are interested in receiving genetic testing–related information through websites, mobile apps, or leaflets [6,12]. We uncovered a wider range of information channel preferences, including interactive technologies (eg, email, patient portals, social media, and smartphone apps), health care providers (through face-to-face conversations, phone calls, and pamphlets), and genetic testing companies and health insurance providers. The inclusion of social media and apps as platforms to receive genetic testing information is a reflection of some patients’ interest in hearing other patients’ experience with genetic testing, suggesting that peer-to-peer patient education, with its potential to be particularly effective in alleviating fears and strengthening patient empowerment, may be integrated with the dominant provider-led education models to deliver genetic testing information to patients.

The differing preferences expressed by the participants seem to suggest that there may be no one-size-fits-all solution to deliver genetic testing–related information. A hybrid model that uses multiple information channels, media, or platforms and delivers information in both clinical settings and beyond may be needed. For example, leaflets may be distributed in clinics to provide basic and simple genetic testing information to patients, whereas a full-fledged interactive website or app may be created to allow patients to access more advanced and detailed information over the course of their cancer treatment. Web-based communities or social media groups may be created to allow patients to exchange genetic testing–related experiences and information. Simultaneously, health care providers such as nurse navigators and hotline nurses may answer patients’ questions by telephone. It is important to note that such solutions should coexist or be integrated with traditional genetic counseling but not replace it.

Similar to most qualitative studies, the results of this study are not quantitatively generalizable in the sense of predicting how many people within a population have certain information needs. However, the rich description of patients’ information needs and their IQ and information delivery preferences outlined in this study will help other researchers determine whether the findings are transferable or can be extrapolated to populations with proximal similarities [56]. Toward these ends, the results should be interpreted with the characteristics of the study sample in mind. First, the sample consisted only of women who had undergone genetic testing. The perspectives of women who have not taken genetic tests may provide insights into information gaps experienced by a broader range of patients with OC and shed light on the reasons why genetic testing was not undergone. Second, most of the participants were White and well-educated. Future studies should attempt to include more minority and underrepresented women. Furthermore, the sample did not involve family members, who often serve as delegates to seek information in cancer care [57]. For genetic testing in particular, many patients avail of testing for the sake of their family members [58]. Therefore, understanding family members’ information needs may be valuable for intervention design.

### Conclusions

Patients with OC have a need for information on various genetic testing–related topics. Genetic counseling alone does not address all of these needs. Interventions that supplement existing genetic counseling are needed. Successful interventions should offer relevant, concise, easy-to-understand, and well-organized (eg, tables and bullet points) information and be available at times and locations needed. Moreover, the information should be appropriate and sympathetic to the cognitive and emotional states of patients with cancer. The patients’ preferences for channels or platforms to receive information differed. A hybrid multichannel information delivery model that combines both health care provider–led and peer-to-peer patient education efforts may be most effective in delivering genetic testing–related information to patients with cancer. Future efforts are needed to explore the feasibility of the multichannel information delivery model and its effectiveness in promoting awareness and acceptance of genetic testing among patients and family members and in empowering them in cancer treatment and care.

## Appendix

Multimedia Appendix 1 Interview and co-design session guide.

## Abbreviations

BRCA: breast cancer gene
CSN: Cancer Survivor Network of the American Cancer Society
IQ: information quality
OC: ovarian cancer
UT: University of Texas
