# Supplementary material for: Understanding the Information Needs of Patients With Ovarian Cancer Regarding Genetic Testing to Inform Intervention Design: Interview Study
Source: JMIR Cancer. 2022 Feb 8;8(1):e31263. doi: 10.2196/31263 (PMC8864522; doi:10.2196/31263)
Supplement: Multimedia Appendix 1 [file cancer_v8i1e31263_app1.docx]

**Interview questions:**

Section I: Basic information

- When were you diagnosed with ovarian cancer? Can you also tell us where you were diagnosed (State and city names will be sufficient)?
- Have you had a genetic testing? When did you have your test? Was it before or after your diagnosis?
- Why did you decide to take the test (motivations)?
- Can you tell us the process through which you took the test?
- How did you think and feel about the test at the time?
- Did you inform your family members of the test? How did your family members think about and react to it?

Section II: Before and during the test

- What did you know about genetic testing when you were about to take the test? What challenges did you face? What information did you want to have at the time?
- What information did you receive from your healthcare providers?
- Did you search any information about genetic testing before taking it? Which sources (e.g., healthcare providers, the internet, family and friends) did you go to? How did you go about the searches? Did you find anything useful?
- Have you had any genetic testing counseling before taking it? If yes, where did you have it? What was the process? How did you feel about the counseling?
- How did you choose your genetic test service vendor(s)? Did you have any difficulties when choosing the service(s)?
- Thinking about the process through which you had the test, how did you feel about your own experience? Anything particularly good, bad, or unforgettable?

Section III: After the test

- Did you have difficulties in understanding the test results? If yes, what were the difficulties?
- Did your doctors interpret the test results for you? What were their roles in treating you and what are their specializations? Did nurses help in any way?
- What information did you wish to have to help you understand the test results?
- Did you search for information yourself to help you understand the results? What information did you look for? What sources did you use? How did you go about the searches? Did you find anything useful?
- Did you talk with your family or friends about your test and test results? If yes, what were their reactions and how did you feel about sharing your test results with them?

Section IV: Overall reflections

- What frustrated you the most in your genetic testing experience? What concerned you the most?
- Looking back, what kinds of information and what kinds of support do you think would have helped to enhance your genetic testing experience, from being informed to take the test, to getting the test done, and then to understand and make sense of the test results?
- From whom do you wish to receive the information from? In what formats do you want such information to be in (e.g., text, audio, video…..)? In what forms of technology do you wish to receive the information (e.g., app, website, telephone, brochures……)?
- How do you think about the genetic testing now?

**Co-design session**

This is the mockup of a website intended to provide genetic testing information to ovarian cancer patients and their family members to improve their experience with genetic testing and with the healthcare system in general (the website will be introduced to patients by the clinics at UT medical school). The mockup serves as a starting point for us to gather user needs and requirements for this topic. Thus, we want to hear your feedback about the website based on your own experience. Please tell us thoughts, comments, and recommendations that you have about the site.

1. *Link to the mockup website:* <https://www.figma.com/proto/S8K7O1Y7h0ZfkvJkNHCBiS/Ovian-cancer-and-genetic-testing?node-id=19%3A716&viewport=-260%2C-396%2C0.15908636152744293&scaling=min-zoom>
2. Probing questions, if needed

- What additional information/content do you think should be included on the website?
- What do you think about the content in terms of its language (i.e., understandability and conciseness), usability, and usefulness? What kinds of features that you want the content to have? (e.g., basic, comprehensive…)
- What do you think about the format of information? What format do you prefer to see?
- What do you think of the navigation of the website?
- What do you think of the layout (e.g., color, icon, font size) of the website?
- What do you dislike about the website?
- What do you like about the website?
- Would it have been helpful for you to have had access to the website when you went through genetic testing?
- If you were to access the website, on what kind of devices would you like to do so? [e.g., smartphone, tablet, laptop computer, or desktop computer]
- Do you have any additional suggestions for us to improve the website?
